# Pigeons show how meta-control enables decision-making in an ambiguous world

**DOI:** 10.1038/s41598-021-83406-7

**Published:** 2021-02-15

**Authors:** Martina Manns, Tobias Otto, Laurenz Salm

**Affiliations:** 1 Division of Experimental and Molecular Psychiatry, Department of Psychiatry, Psychotherapy and Preventive Medicine, LWL University Hospital, Ruhr-University Bochum, Bochum, Germany; 2 Department of Cognitive Psychology, Faculty of Psychology, Institute of Cognitive Neuroscience, Ruhr University Bochum, Bochum, Germany; 3 Biozentrum, University of Basel, 4056 Basel, Switzerland

**Keywords:** Neuroscience, Cognitive neuroscience, Decision, Problem solving

## Abstract

In situations where the left and right brain sides receive conflicting information that leads to incompatible response options, the brain requires efficient problem-solving mechanisms. This problem is particularly significant in lateralized brains, in which the hemispheres differ in encoding strategies or attention focus and hence, consider different information for decision-making. Meta-control, in which one hemisphere dominates ambiguous decisions, can be a mechanism that ensures fast behavioral reactions. We therefore confronted pigeons with a task in which two stimulus classes were brought into conflict. To this end, we trained pigeons simultaneously on two categories (cats or dogs) whereby each hemisphere learnt only one of the categories respectively. After learning, the birds were confronted with stimulus pairs that combined a picture with a cat (positive for one hemisphere) and a picture with a dog (positive for the other hemisphere). Pecking responses indicated the hemisphere dominating response selection. Pigeons displayed individual meta-control despite equal categorization performances of both brain hemispheres. This means that hemispheric dominance only emerged in interhemispheric conflict situations. The analysis of response latencies indicate that conflict decisions relied on intrahemispheric processes. Interhemispheric components played a role for more complex decisions. This flexibility could be a crucial building block for the evolutionary success of a lateralized brain.

## Introduction

When animals orient in a natural environment, they are permanently confronted with stimuli appearing in the left and right hemispace, which compete for attention and which can be of differential significance for survival. Therefore, animals must constantly evaluate incoming information for relevance and decide on appropriate behavioral responses. Most bilaterian species process sensory input from one half of the environment on one side of the nervous system, so that the simultaneous emergence of stimuli in the left and right hemispace, which trigger differential or even incompatible response tendencies, can lead to decision conflicts between the two halves of the brain^1^. One basic organization principle to cope with such problems is neuronal lateralization. Lateralization is remarkably widespread in the animal kingdom and can be found in all vertebrate classes^2–4^ but also in many invertebrate species including bees, fruit flies, octopuses or nematodes^5^. In a lateralized nervous system, the two brain sides differ in their structural and functional organization so that both take over different functional roles. Consequently, the hemisphere specialized for a specific function dominates decision-making and response selection. Moreover, lateralization enables coping with two simultaneous tasks, whereby each hemisphere can consider different information from the environment^6–10^. In ambiguous situations however, interhemispheric conflicts can arise when both hemispheres process information in their specialized ways and generate incompatible response options. Since deciding between the conflicting options leads to costly reaction time delays^11,12^, hemispheric lateralization can only be adaptive when neuronal mechanisms minimize interhemispheric interferences and ensure quick and appropriate behavioral responses.

An efficient way to solve these computational problems can be meta-control meaning that one hemisphere constituently takes charge of response selection in case of interhemispheric conflict independent from any hemispheric specialization^13^. Meta-control has been shown for human^13^ and non-human primate brains^14^. However, as indicated above, interhemispheric conflicts because of contradictory environmental cues can be special problem of animals with laterally placed eyes and completely crossed optic nerves^15^. First studies provide hints for the existence of meta-control in chickens^11^ and pigeons^16–19^, that is, in species, which display structural and functional asymmetries of their visual systems^1,3,4,20,21^. This lateralization on the other hand makes it likely that the observed hemispheric dominances are caused by hemispheric specializations. In pigeons for instance, meta-control studies have been based on color discriminations^16–19^. Since studies report a left-hemispheric superiority for color discrimination under several task conditions^17,22,23^, observed dominance pattern may reflect hemispheric processing asymmetries and not necessarily meta-control.

The neuronal mechanisms mediating meta-control are not well understood but suggested models rely on intra- or interhemispheric processes^13,24–26^. According to the horse race model^25^, a speed contest between the two halves of the brain decides which hemisphere consistently wins. This means that meta-control is the result of differential processing times of intrahemispheric neuronal operations and the dominant hemisphere just gains quicker control over descending premotor networks^13,24–27^. Meta-control, on the other hand, is not strictly fixed and factors like task instructions, information processing strategy or computational complexity influence which hemisphere controls decision between incompatible response options^26^. This in turn suggests that meta-control is controlled by commissural fiber systems, which enable interhemispheric communication and information matching at a higher level of processing^13,28^. In pigeons, both mechanisms can play a role when response selection is based on associative value of conflict stimuli after color discrimination training^17–19,22^. It is however, completely unknown whether and which mechanisms play a role for decisions requiring higher cognitive coding. We therefore investigated meta-control in a conflict task based on categorization.

**Figure 1:**
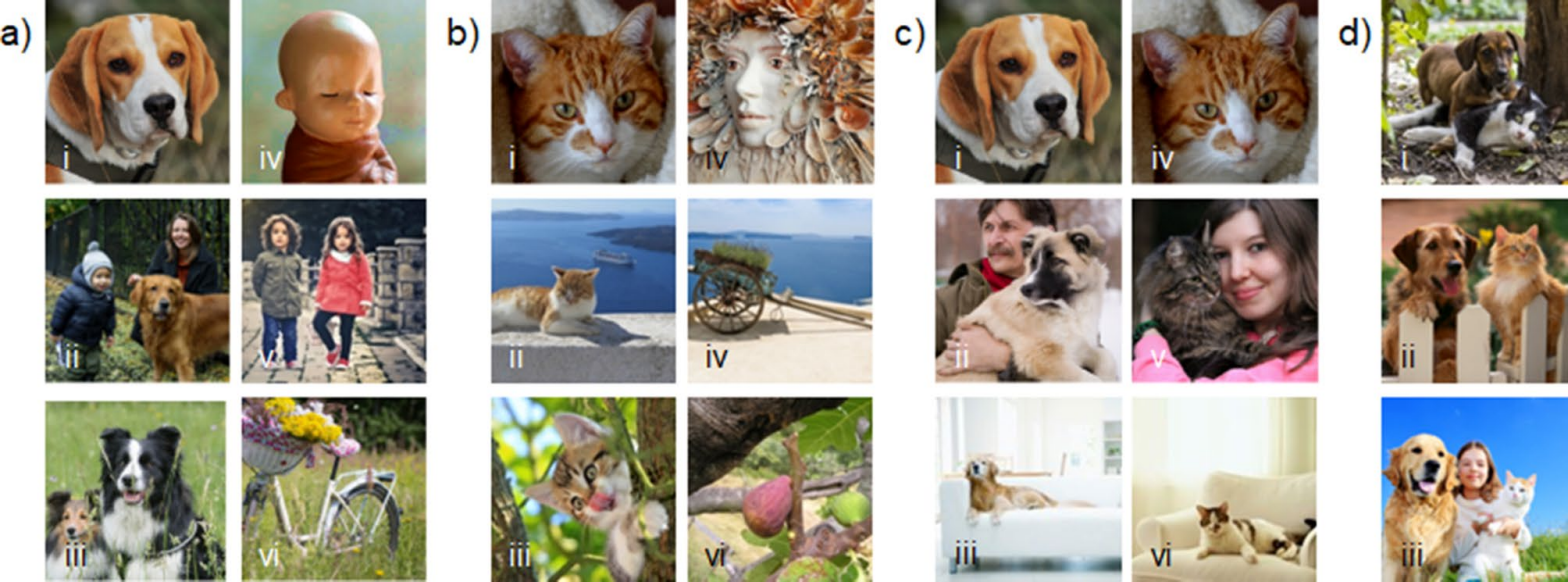
Examples of pictures used illustrating differential, complex and comparable composition of the different stimulus types: (**a**) exemplary S+, S− of the dog category; (**b**) exemplary S+, S− of the cat category; (**c**) exemplary conflict stimulus pairs, which can be composed of trained (i–iv) or untrained (ii–v, iii–vi) items; (**d**) exemplary Super S+ presenting cats as well as dogs (royalty free pictures; photo credits: a.i Jose Somovilla on Pixabay; a.ii Ksenia Chernaya on Pexels; a.iii Gerhard Bogner on Pixabay, a.iv joe puengkaew on Pixabay; a.v Bess Hamiti on Pexels; a.vi Kristine Lejniece on Pixabay; b.i LILO on Pixabay; b.ii tsefan cxhang on Pixabay; b.iii Kessa on Pixabay; b.iv Martina Bulková on Pixabay; b.v didiwo on Pixabay; b.vi Simon Steinberger on Pixabay; c.ii alatyren on Pixabay; c.iii bloom images on getty images; c.v Uschi Dugulin on Pixabay; c.vi swll media on getty images; d.I Ilona Ilyés on pixabay; d.ii fuse on getty images, d.iii skynasher on getty images).

Categorization is a perfect tool for investigating meta-control, since successful categorization results from higher order analysis of visual stimuli^29,30^ and because the two hemispheres differ in their preferred encoding strategy. While the left brain side tends to adopt a category-based strategy, the right one favors consideration of memory formation^31^. Since pigeons are well known for their excellent categorization abilities^22,32–34^, a large set of possible stimulus classes is available to develop a task putting two classes in conflict. We decided for the categories ‘dog’ and ‘cat’ since both display similar complexity and can be learnt equally well^35^. This should minimize decisions based on class-inherent characteristics. We trained our pigeons in discriminating pictures displaying dogs or cats, respectively from similar pictures without the category-defining animals (Fig. 1a, b) whereby each hemisphere learnt only one of the stimulus classes. After training, the pigeons were confronted with conflict stimulus pairs, each combining a positive item from the dog and cat category (Fig. 1c). Consequently, the two hemispheres should choose opposite stimuli to peck on. Response patterns could indicate the hemisphere taking charge of response selection and differential reaction times could provide hints for the underlying neuronal mechanisms.

## Results

### Monocular training and categorizing

In a first step, the pigeons had to learn two stimulus classes (cats or dogs)—one with each eye/hemisphere (Fig. 1a, b). To this end, they were trained in daily sessions with alternating eye-conditions to discriminate between pictures with (S+) and without (S−) cats or dogs, respectively. For each trial of one session, a pair of stimuli consisting of randomly selected S+ and S− were presented and pecking onto a S+ was rewarded with food. Pecking onto an S− was punished by turning off the light within the Skinner box for four seconds (for experimental details, see Methods section, Tables S4, S5). When the animals reached the learning criterion of more than 80% correct choices on two consecutive sessions with each eye, probability of food reward was gradually decreased from 100 to 40% to avoid extinction of responses during test sessions when critical trials were neither rewarded nor punished.

**Figure 2:**
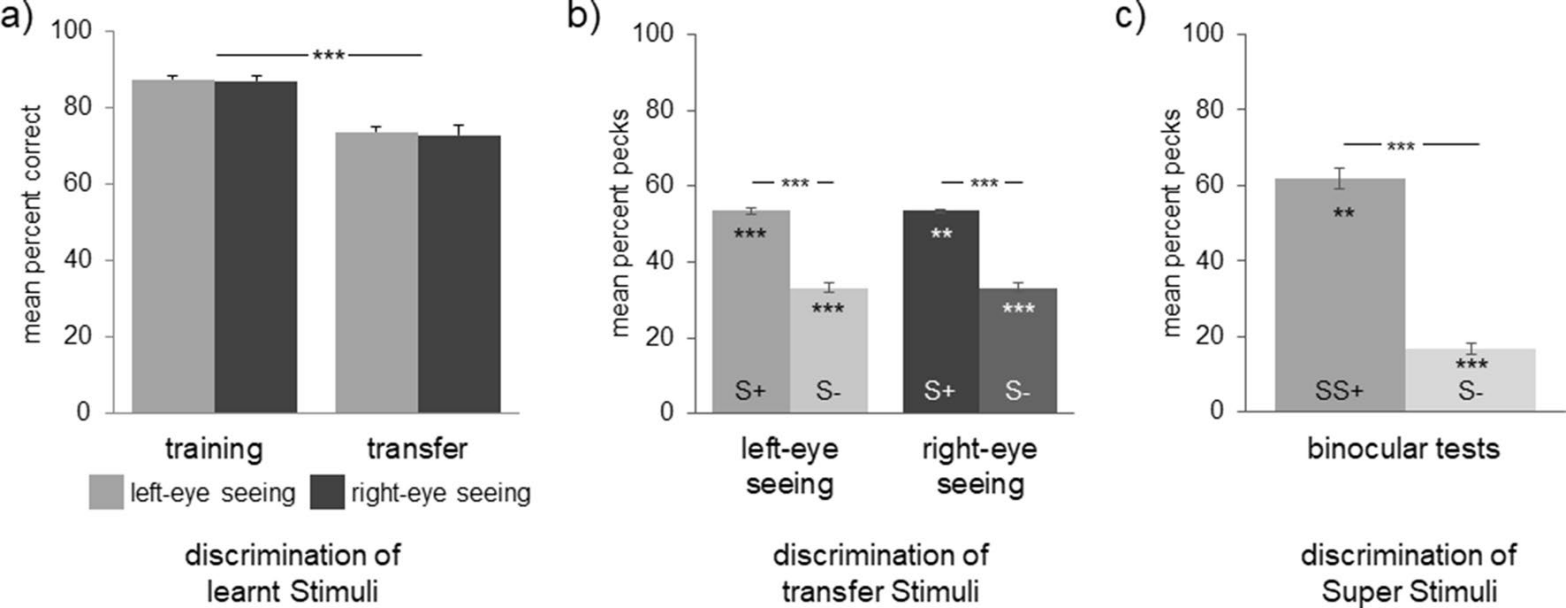
Discrimination of the stimulus classes—(**a**) Discrimination accuracy of learnt stimuli during training and transfer tests expressed as the percentage of trials responded correctly; (**b**) percent pecks onto positive (S+) and negative (S−) untrained stimuli during transfer tests; (**c**) percent pecks onto positive (SS+) and negative (S−) stimuli during binocular conflict tests. SS+ stimuli include at least one dog and one cat and are hence, positive for both hemispheres. Bars represent standard error (**p < 0.01, ***p < 0.001 according to t-tests for dependent samples or one sample t-tests).

Pigeons required a mean 142 ± 15 (varying between 34 and 220) sessions until reaching the final learning criterion. There was neither a difference between the eye conditions (left eye: 71 ± 27; right eye: 72 ± 26) nor between the two stimulus classes (cats: 72 ± 26; dogs: 70 ± 27). There was also no difference in discrimination accuracy at the end of training (mean of the last two sessions) between the eyes (Fig. 2a) or between the two categories (cats: 87% ± 5%; dogs: 88% ± 3%). Only one pigeon (T603) never reached the learning criterion with the left eye (73%). This pigeon nevertheless passed the transfer test (see below) with the left eye and was therefore included into all analyses.

After training, we proved, whether the pigeons successfully acquired the two categories. To this end, the pigeons were tested in a modified version of the training program—still under monocular seeing-condition. In this version, trials with trained S+ and S− were interspersed with trials presenting new S+ and S− (Tables S4, S5). Decisions for the novel positive items indicated transfer to previously unknown exemplars of the learned stimulus sets, which is taken as evidence of successful categorization^29^.

During transfer tests, discrimination performance with both eyes decreased significantly for the learnt training stimuli (t-test for dependent samples: t = 8.235, *p* = 0.000, Fig. 2a). A decrease in the discrimination accuracy for the learnt stimuli has also been reported in other studies and could be explained by a higher complexity of the test situation, in which not only the known, but also new stimuli were presented^36–38^. When confronted with new positive (S+) and negative (S−) stimuli, the pigeons decided significantly more often for the new S+ compared to new S− (t-test for dependent samples: t = 16.175, *p* = 0.000). Pigeons did not respond in about 14 ± 3 trials per session, but percent decisions for new S+ were significantly above and for new S− significantly below 50% chance level (one sample t-tests: *p* = 0.0000 for both stimuli types; Fig. 2b). There were no differences in transfer performance between either the eye conditions or the two stimulus classes. Thus, discrimination as well as transfer tests indicated that the two hemispheres learned, memorized and categorized equally well and hence, simple associative processes could not explain any hemispheric dominance observed during conflict choices.

### Binocular conflict tests

After the two monocular tests were completed, the conflict tests sessions begun. During these tests, the pigeons could see with both eyes while confronted with ambiguous stimulus pairs composed of a picture with a cat (positive for one hemisphere) and of a dog (positive for the other hemisphere (Fig. 1c; Tables S4, S5). Within single sessions, four types of conflict appeared: (**a**) trained cat—trained dog, (**b**) untrained cat—untrained dog, (**c**) trained cat—untrained dog, (**d**) untrained cat—trained dog. Stimuli were combined to fixed pairs to ensure similar composition and complexity of the presented pairs (Fig. 1c). Pecking onto one of the presented items was neither rewarded nor punished. To maintain motivation during the test sessions, the pigeons had to discriminate superstimuli (SS+), which depicted both cats and dogs (Fig. 1d), from new S−. The SS+ were correct for both hemispheres and could be rewarded without biasing conflict responses.

In 60% of the trials within single sessions (Table S4), the pigeons had to discriminate SS+ from S−. In about 21 ± 3% of the trials, the pigeons did not respond. When pecking, pigeons decided significantly more often for the new SS+ compared to new S− (Fig. 2c; t-test for dependent samples: t = 13.977, *p* = 0.000). A high level of correct decisions was already acquired during the first two sessions supporting successful categorization of the stimulus classes (t-test for dependent samples: t = 12.824, *p* = 0.000; Fig. S1a).

**Figure 3:**
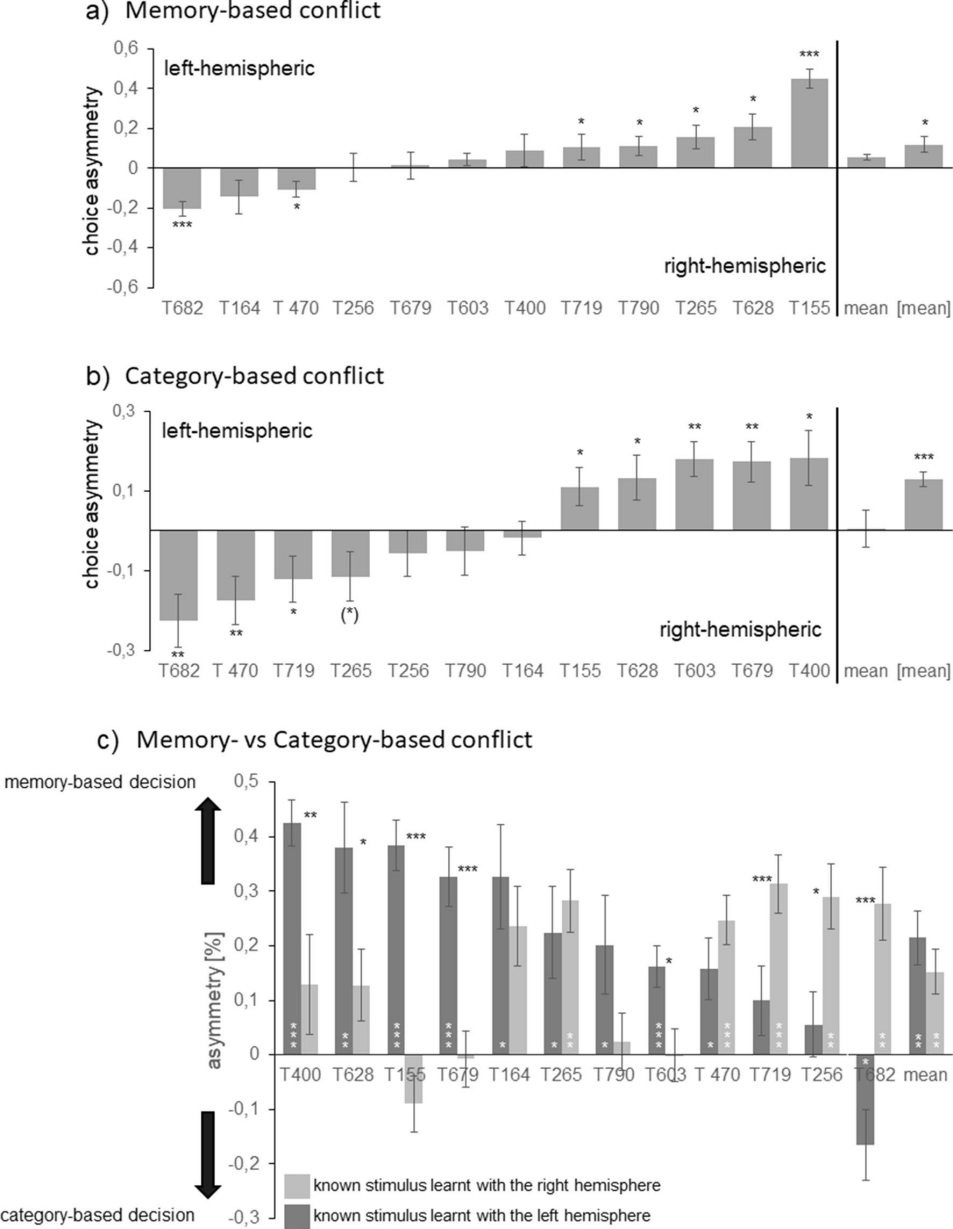
Decision asymmetry for different conflict types—(**a**) memory-, (**b**) category-based conflicts (numbers represent single pigeons): positive values indicate left- and negative values right-hemispheric dominance. Absolute values ([mean]) but not mean decision asymmetries of all pigeons differ significantly from zero. (**c**) Memory- versus category-based conflicts: positive values indicate a memory-, negative values a category-based decision dominance. In mean, the pigeon group decides for the known stimulus but degree of left- and right-hemispheric memory-based decision dominance differs individually (black asterisks). Bars represent standard error (*p < 0.05; **p < 0.01, ***p < 0.001 according to t-tests for dependent samples or one sample t-tests).

Interspersed in trials presenting SS+/S− stimulus combinations, the pigeons were confronted with the different conflict types. Independent from conflict type, the pigeons did not respond in about 20% of the trials/session. In 10% of the trials (Table S4; Fig. 1c), pairs of two trained stimuli appeared—one learnt with the left and one learnt with the right hemisphere. Thus, pecking onto one of the items was a memory-based decision presumably controlled by the hemisphere that had learnt the responded stimulus. Most pigeons chose stimuli from one of the two stimulus classes more often and this dominance was significant for seven of the birds (Fig. 3a). Since however, some pigeons displayed left- and others right-hemispheric dominance, mean decision asymmetry did not differ from zero (one sample t-tests: t = 0.841, *p* = 0.418), while absolute asymmetry value did (one sample t-tests: t = 3.557, *p* = 0.004; Fig. 3a).

In further 10% of the trials (Table S4), the pigeons were confronted with pairs of untrained stimuli from both categories (Fig. 1c). Accordingly, the hemispheres had to categorize when deciding for one of them. Comparable to the memory-based conflict decisions, most (nine) pigeons displayed individual hemispheric dominance meaning that the birds decided significantly more often for the left- or right-hemispheric stimulus, respectively (Fig. 3b). While mean decision asymmetry did not differ significantly from zero (one sample t-tests: − 0.032, t = , *p* = 0.975), absolute values did (one sample t-tests: t = 7.014, *p* < 0.00002; Fig. 3b).

In 20% of the trials, combinations of trained/untrained stimuli constituted the conflict pairs (Table S4). In this case, one hemisphere had to memorize and the other one had to categorize when deciding for one of the stimuli. In general, the pigeons decided more often for the trained stimulus irrespective for which hemisphere it was correct (t-tests for dependent samples: *p* < 0.01 for left- and right hemispheric decisions; Fig. 3c). Asymmetry of decisions and hence, advantage for the memorized stimuli did not differ between left- and right-hemispheric responses (t-tests for dependent samples: t = 0.793, *p* = 0.445) though at individual level, extent of decision asymmetry varied (Fig. 3c). Intriguingly, percentage of hemispheric-specific choices correlated significantly between all conflict types (Table S1). This means that the more one hemisphere dominated memory-based decisions, the more this hemisphere controlled conflict-based decisions or outvoted the memorizing one in a memory- versus category-based conflict. Dominance in conflict decisions was however, independent from discrimination or transfer performance as indicated by the absence of any correlation between conflict decisions pattern and monocular training or transfer performance (Table S2).

### Response latencies

The analysis of reaction times can provide important insight into the neuronal mechanisms mediating hemispheric dominance^18,19,22^. We therefore compared response latencies for the different choice types during binocular conflict choices.

**Figure 4:**
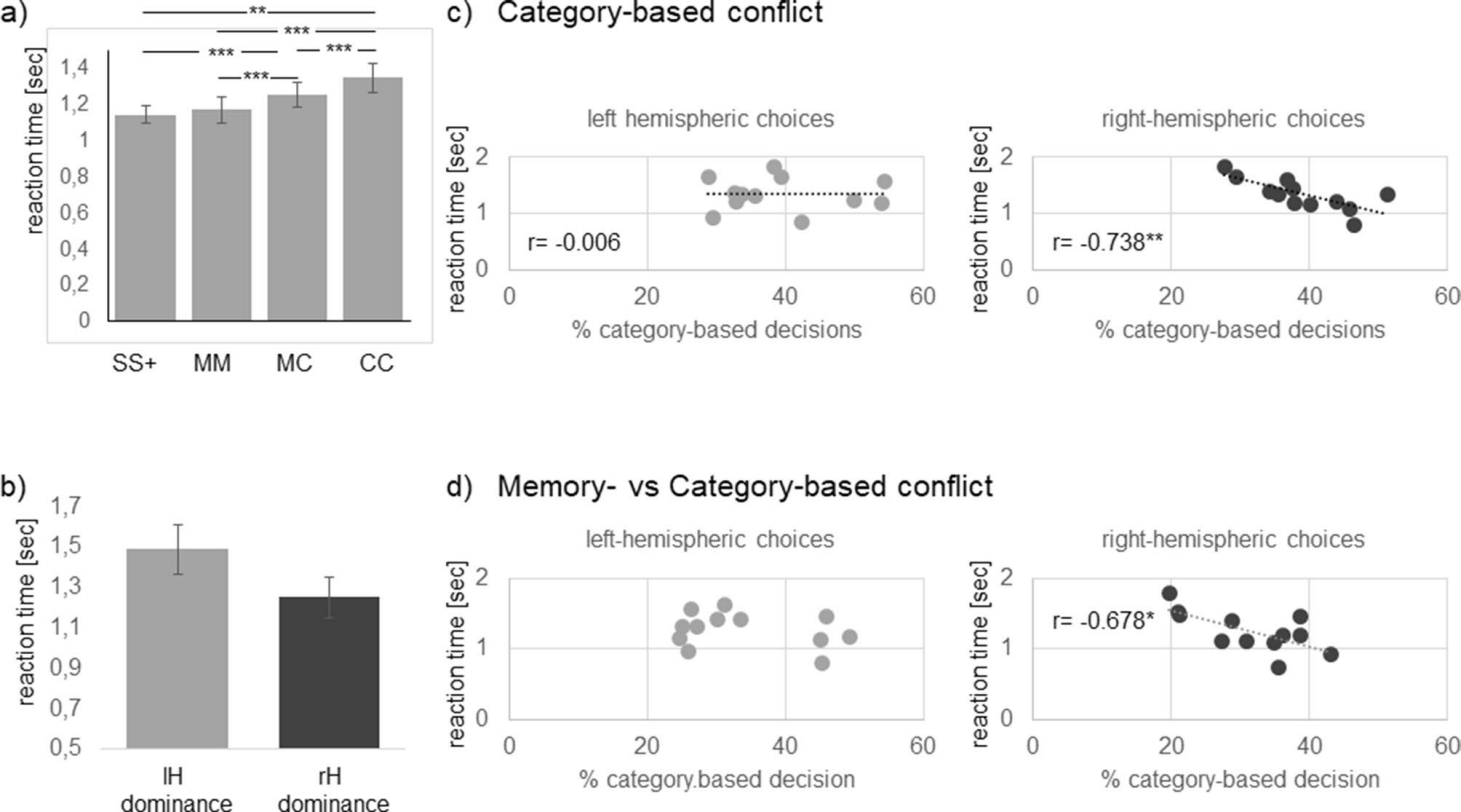
Analysis of reaction times—(**a**) Response latencies for pecking onto SuperS+ (SS+) and conflict choices during binocular conflict tests (*MM* Memory-based; *CC* Conflict-based; *MC* Memory- versus Catory-based); (**b**) mean reaction time of pigeons displaying left—(lH) or right—(rH) hemispheric decision dominance for category-based decisions; (**c**), (**d**) relations between percentage of category-based decisions and response latencies in different conflict types. Bars represent standard error. (*< 0.05, **< 0.01, ***< 0.001 according to t-tests for dependent samples or Pearson correlations).

Pigeons displayed fastest responses when pecking onto SS+ (Fig. 4a), response latencies for new S− were significantly longer (1.22 ± 0.24 s (s); t-test for dependent samples: t =  −2.711, *p* < 0.05). Response latencies of left- and right-hemispheric choices did not differ and we therefore firstly compared mean reaction times for memory-, category-, and memory- versus category-based conflicts and pecks onto SS+. A 4× MANOVA showed that reaction times differed depending on stimulus type (F (3, 33) = 11.279, *p* < 0.00003, partial eta squared = 0.506). Memory-based conflict decisions were as fast as pecking on SS+ while response latencies to category-based decisions were significantly prolonged. Response latencies for memory- versus category-based conflicts displayed intermediate length (Fig. 4a). These differences primarily indicate that categorization enhanced reaction times. Accordingly, during the first two sessions when the pigeons saw the SS+ for the first time and therefore had to categorize for discrimination, reaction times were longer and did not differ from that of category-based conflict decisions (Fig. S1). The required analysis strategy was however, not the only determinant for response latencies as indicated by the memory- versus category-based decisions. In most cases, pigeons decided for the memorized stimulus and hence, we could expect reaction times comparable to the ones for memory-based conflict decisions. Actually, reaction times were significantly longer than memory—though shorter than that for category-based conflict decisions (Fig. 4a).

Since each pigeon displayed left- as well as right-hemispheric choices for each conflict type, we correlated percent hemispheric-specific decisions with reaction times. For memory-based conflict decisions, no consistent correlations could be estimated. This was different for category-based decisions (for details see Table S3a,b). Intriguingly, percentage of right-hemispheric choices for category-based decisions was negatively correlated with reaction time for untrained conflict types (Pearson correlation: r =  −0.738, *p* < 0.01) while left-hemispheric decisions were independent from reaction times (Pearson correlation: category-based decisions r =  −0.006, *p* = 1; Fig. 4c). In accordance with this difference, pigeons displaying right-hemispheric dominance for category-based decisions reacted faster than pigeons displaying a left-hemispheric dominance (Fig. 4b). In a similar way, right- but not left-hemispheric response latencies correlated negatively with category-based decisions in a memory- versus category-conflict. Of note, it is interesting that the percentage of left-hemispheric choices seemed to be clustered with one group displaying 25–34% and one group displaying 45–50% left-hemispheric choices. While the second group only included pigeons with a left-hemispheric choice dominance, decision dominance was mixed for the first group. Because of potential clustering we did not conducted a correlation analysis with this data set (Fig. 4d).

## Discussion

Our data provide clear evidence for meta-control in the pigeon brain when confronted with conflict choices based on the discrimination of complex stimulus classes. Analysis of reaction times suggests that hemispheric dominance is primarily based on intrahemispheric processes but includes interhemispheric components in more demanding decisions.

Virtually all pigeons passed training and transfer tests with each eye. This was not surprising since pigeons are able to categorize a wide variety of perceptual categories^29,31–34,39,40^. Although transfer performance was relatively low compared to the discrimination of comparable stimulus classes^31,41^, the birds decided significantly for the new positive items during transfer tasks. This indicates successful categorization and was further supported by discrimination of the SS+ presented during binocular conflict tests. Already during the first two sessions, the pigeons correctly transferred discrimination to these novel stimuli. This implies that the pigeons could use a memory- as well as category-based strategy for response selection in our experiment. For interpreting the observed conflict choice pattern as meta-control, three observations are critical. First, there was no difference in learning and transfer performance between the cat and dog category indicating comparable difficulty of both stimulus classes. Therefore, decisions should in principle not have been determined by different category complexity. Second, we did not observe any difference in learning speed, discrimination accuracy or transfer performance between the hemispheres (neither at the population- nor at the individual level) indicating equal categorization abilities of both brain sides. Hemispheric differences only arose in ambiguous situations when two positive stimuli were put into conflict. Third, the percentage of hemispheric-specific conflict decisions did not correlate with monocular training or transfer performance. Thus, dominance of one hemisphere does not reflect superior processing of one brain side but presumably depends on specific conflict solving mechanisms^24^.

When confronted with an ambiguous stimulus pair, each hemisphere presumably pays attention to the item belonging to the learnt stimulus class and therefore, the hemispheres should each decide for a different item*.* This process evaluates and decides individually for each pair of stimuli, presumably dependent on their specific visual characteristics. Similarity of the stimulus classes and a wide range of picture styles, displayed sceneries, and cat or dog breeds however, should have led to a balanced decision pattern in favor of the left and right hemisphere. Nevertheless, in most of our pigeons, one of the hemispheres significantly dominated response selection. For some pigeons, the left, for others the right brain side controlled the conflict decisions. Thus, meta-control was not aligned in the same direction at group level. We cannot directly identify causes for left- or right-hemispheric dominance but it is conceivable that differential direction and degree of lateralized perceptual, premotor and/or cognitive processes result in individual meta-control pattern.

Additionally, the required analysis strategies could influence hemispheric decision dominance^13,26^. The two hemispheres of the pigeon brain differentially encode conflicting associative memory from the contralateral brain side^37^. When discriminating the complex perceptual category “human”, the left hemisphere preferentially relies on categorization while the right brain side favors a memory-based decision^31^. We therefore expected a right-hemispheric dominance when conflict decisions were based on memorization and a left-hemispheric dominance for choices requiring categorization. We however, did not detect any strategy-dependent differences in decision asymmetry at group level. We only observed dominance of memory-based decisions in memory- versus category-based conflicts. This can be simply explained by faster processing of trained items leading to an advantage of the memorizing hemisphere (see also below). Nevertheless, in several trials the categorizing hemisphere overruled the memorizing one. The more dominant a hemisphere was in such trials, the more dominant it was in purely memory- or category-based decisions. This observation further supports the presence of specific meta-control mechanisms in the pigeon brain. Dominance of the same hemisphere independent from conflict type however, can be based on different neuronal mechanisms. This is supported by the analysis of reaction time data.

Models for the neural basis of meta-control assume the involvement of intra- and interhemispheric processes to different degrees. The analysis of response time patterns can provide important clues for their specific contributions^18,19,22^. The intrahemispheric race model^25^ suggests that both hemispheres start to process information independently and the winning hemisphere is the one faster accessing descending motor circuits. Consequently, reaction times of conflict decisions should not differ from non-conflicting ones^22,25^. Interhemispheric models, on the other hand, suggest that commissural interactions mediate the critical mechanism that leads to the dominance of one hemisphere. This additional processing step should extend the response times^24^. Analysis of response latencies in our conflict task firstly showed that mean reaction time did not differ between left- and right hemispheric decisions. This underlines that neither of the hemispheres had a principle processing advantage. When comparing response latencies for discrimination of SS+ (no conflict) and the different ambiguous stimulus pairs, we did not observe a difference in response latencies between pecks onto SS+ and memory-based conflict choices. Thus, a conflict decision did not enhance reaction time, which in turn supports the intrahemispheric race model. It is likely that asymmetrical speed and/or efficiency of intrahemispheric top-down mechanisms yield unilateral dominance over the course of processing^17,19,22^. When confronted with category-based decisions, the pigeons needed longer to peck compared to the discrimination of SS+ but also compared to memory-based conflict decisions. It is therefore likely that the more demanding computational processes underlying categorization increased response latency^29^ but not the actual decision conflict. This conclusion is supported by the decreasing reaction times over the test sessions when pecking on the SS+.

Response latencies for memory- versus category-based decisions however, indicated that a sole intrahemispheric process is not sufficient to explain all aspects of conflict decision-making. As already discussed above, pigeons decided more often for the trained items. Consequently, the memorizing hemisphere should had have a processing advantage and we could have expected response times that corresponded to those of purely memory-based conflict decisions. This was however, not the case. Response latencies were enhanced though faster than for category-based conflict responses. This indicates that the memorizing hemisphere determined the response but only after an additional neuronal step, which eventually considered contralateral information processing. Exactly such a two-step process is suggested in a recent study of Xiao & Güntürkün^22^. Primarily, one hemisphere in the pigeon brain (typically the left one) is faster in processing visual information and therefore controls pecking responses according to the speed contest model. In addition, the left brain side is able to modify activity within the contralateral right hemisphere via the anterior commissure, which connects the two arcopallial premotor areas of the pigeon’s brain. Xiao and Güntürkün suggest that under conditions of interhemispheric competition, left arcopallial neurons could delay the spike time in the right hemisphere. As a result, the neurons of the right hemisphere come too late to control a response and the left hemisphere governs decisions. This model therefore suggests that dominance of the left and right hemisphere emerges from different neuronal mechanisms. While generation of left-hemispheric meta-control may include interhemispheric components, right-hemispheric dominance must result purely from fast intrahemispheric processes. Correlations between percent hemispheric-specific decisions and response latencies for different conflict decisions supported these assumptions but in a conflict-type dependent manner. Memory-based decisions were generally independent from reaction time. When a conflict decision required categorization, percent left-hemispheric decisions were unrelated to reaction times, too. A closer look into the data set indicated clustering of left-hemispheric choices with one group displaying a high percentage and one group with a low percentage of left-hemispheric choices. However, the number of experimental animals was too low to confirm clustering and therefore, we cannot exclude that this pattern only occurred by chance.

In contrast, right-hemispheric decisions correlated negatively with reaction times. In a similar way, right- but not left-hemispheric response latencies correlated negatively with category-based decisions in a memory- versus category-conflict. This means that the right hemisphere could only take control over response selection when it was especially fast. In accordance with this difference, pigeons displaying right-hemispheric dominance for category-based conflict decisions reacted in mean faster than pigeons displaying a left-hemispheric dominance. Overall, interrelations between hemispheric-specific choices and reaction times suggest that primarily intrahemispheric mechanisms govern which hemisphere controls behavioural output. When the hemispheres are forced to adopt different information processing strategies (in our case: memorizing or categorizing), the impact of interhemispheric processes may increase. This exemplifies a flexible recruitment of hemispheric resources depending on task complexity or situational context^26^. This idea however, must be tested in studies, which systematically modify left- or right-hemispheric processing operations.

Flexible meta-control represents an efficient way to cope with ambiguous situations allowing efficient decision-making and fast behavioral responses. Thereby, differential direction of individual meta-control as it was also observed in previous studies^16,18,19^ may support phylogenetic success. Recently, several publication discuss that fluctuating lateralization might be abundant in natural populations to reduce predictability of behavior and therefore provides a fitness advantage^42–44^. In this regard, individual meta-control may contribute to unpredictable behavior responses differentially integrating left- and right-hemispheric processes and thereby, increases fitness advantages of an asymmetrical brain.

## Methods

### Subjects

Twelve unsexed adult pigeons (*Columba livia*) obtained from local breeders were used in this study. The birds were housed partly as a group (ten pigeons) within one aviary, or within single cages (pigeons 155, 603). All were maintained on a 12-h light–dark cycle. Water and grit were freely available while food was restricted to keep the weight at 75–85% of free-feeding weight. Food was provided daily during training and after the sessions when necessary. Each bird conducted one session per day under different seeing conditions depending on the experimental phase. To restrict view during monocular sessions, rings of Velcro (soft part) were fixed around the pigeons’ eyes three to four days before training started using non-toxic glue (UHU Crafts glue). In this way, cardboard caps with the Velcro hook counterpart glued on the inside could be easily attached and removed^19,37^. The experiments were carried out in compliance with the European Communities Council Directive of September, 22 2010 (2010/63/EU) and the specifications of the German law for the prevention of cruelty to animals and was approved by the animal ethics committee of the Landesamt für Natur, Umwelt und Verbraucherschutz (LANUV) NRW, Germany. We confirm that all methods were carried out in accordance with relevant guidelines and regulations and that the study was conducted in compliance with the ARRIVE guidelines.

### Apparatus

Pigeons were trained and tested in a custom-made operant chamber (35 × 39 × 39 cm^3^). Two LED strips illuminate the chamber, which were turned off as mild punishment during training. Stimuli were presented on a TFT LCD touchscreen monitor (model ET1515L with APR technology, Elo Touch Solutions, Inc., Milpitas, CA, USA, with 1024 × 768 resolution) fixed to the back of the chamber. Centrally below the touch screen, a food hopper delivered mixed grain as reward. As a secondary reinforcer, the feeder was illuminated when food was presented. Experimental sessions were controlled and recorded by custom-written MATLAB programs (MathWorks, Natick, MA, USA) using the Biopsy Toolbox^45^.

### Stimuli

The stimuli were digitized jpeg files and selected from different royalty-free photo libraries (getty images, pexels, pixabay; Fig. 1). They were presented 5 × 5 cm^2^ (142 × 142 pixels, 72 dpi) on the monitor. All stimuli showed complex natural scenes eventually including humans, objects, and/or animals. Positive stimuli (S+) were characterized by the presence of dogs or cats, respectively, which could vary in number, size, orientation, location or breed. Negative stimuli (S−) displayed similar sceneries just without cats or dogs. For training trials, 800 pictures, 400 of each class, were used. Positive and negative pictures were separately grouped into four parallel sets of 50 positive and 50 negative pictures. Training sets were counterbalanced among the subjects and between the hemispheres (according to^31,41^).

Further S+ and S− were selected for transfer and binocular conflict tests (Table S4). In addition, pictures depicting both cats as well as dogs served as superstimuli (SS+) during the binocular conflict tests (Table S4).

### Training and testing procedure

After bi- and monocular autoshaping^18^, category training phase began whereby the birds learnt to discriminate two stimulus classes—one with each eye (Table S4,5). Pigeons were trained in daily sessions (five days a week) with alternating eye-conditions.

Pigeons learnt to discriminate between pictures with and without cats or dogs, respectively in a forced choice paradigm (Fig. 1a, b). For each trial of one session, a pair of stimuli consisting of randomly selected S+ and S− were presented. The start of each trial was indicated by a short tone and the pigeons had to peck once on a white initialization key within a 4 s time window. Then, one stimulus pair was presented for a fixed time interval of 8 s and had to be pecked once. When a correct choice was made, the display disappeared and eventually (in 40–100% of the trials depending on the experimental phase) reward was given (2–4 s access to food and feeding light on); incorrect choices were mildly punished by 4 s light time out. In case of false responses, the same stimulus pairs were presented until correct choices were made but no more than 10 times in a row. The next trial followed an 8 s inter-trial interval (ITI). After reaching the learning criterion of > 80% correct choices two times in a row with either eye, transfer tests started. This test was similar to the monocular training program, but trials showing learned training stimuli were interspersed with 20% trials, which confronted the pigeons with new positive (S+) or negative (S−) examples of the stimulus classes (Tables S4, S5). Responses to test stimuli were not rewarded. After finishing the transfer tests, pigeons were retrained to learning criterion. Then conflict test phase started. Thereby, 4–6 binocular test sessions (300 trials per session) alternated with monocular training sessions.

Conflict sessions consisted of 40% trials displaying a conflict stimulus pair (Table S4, S5, Fig. 1c) interspersed into 60% trials presenting SS+ and SS− (Fig. 1d). Stimuli were combined to fixed pairs whereby each pair was presented potentially twice during one session each with reversed top-down position. Decisions for one of the conflict stimuli were neither punished nor rewarded. Pecking stopped stimulus presentation and the next trial started after 8 s ITI.

### Analysis

Statistical analysis was conducted with IBM SPSS Statistics 21. We analyzed percent pecking decisions (in relation to initialization rate) within single sessions. Choice asymmetry was calculated as (left-hemispheric decision—right-hemispheric decision)/(left-hemispheric decision + right-hemispheric decision). When describing analyzed data, we report means along with standard deviation as a measure of variability. Normal distribution was evaluated by Kolmogorov–Smirnov and Shapiro–Wilk tests and homogeneity of variance by Levene as well as Brown-Forsythe-tests. We conducted two-tailed parametric tests (t-tests for dependent samples, MANOVA), and Pearson correlations.

## Supplementary Information

Supplementary Information.

## Data Availability

The data that support the findings of this study are currently available from the corresponding author upon request. A data overview is currently uploaded at https://doi.org/10.6084/m9.figshare.13102718.v1.
