# Supplementary material for: Pigeons show how meta-control enables decision-making in an ambiguous world
Source: Sci Rep. 2021 Feb 15;11:3838. doi: 10.1038/s41598-021-83406-7 (PMC7884740; doi:10.1038/s41598-021-83406-7)
Supplement: Supplementary file 1 — Supplementary Information. [file 41598_2021_83406_MOESM1_ESM.pdf]

Supplementary Information for

“Pigeons show how meta-control enables decision-making in an ambiguous world”

Martina Manns, Tobias Otto & Laurenz Salm

Corresponding author: Martina Manns
Email: martina.manns@rub.de

This PDF file includes:

Figure S1
Tables S1 to S5

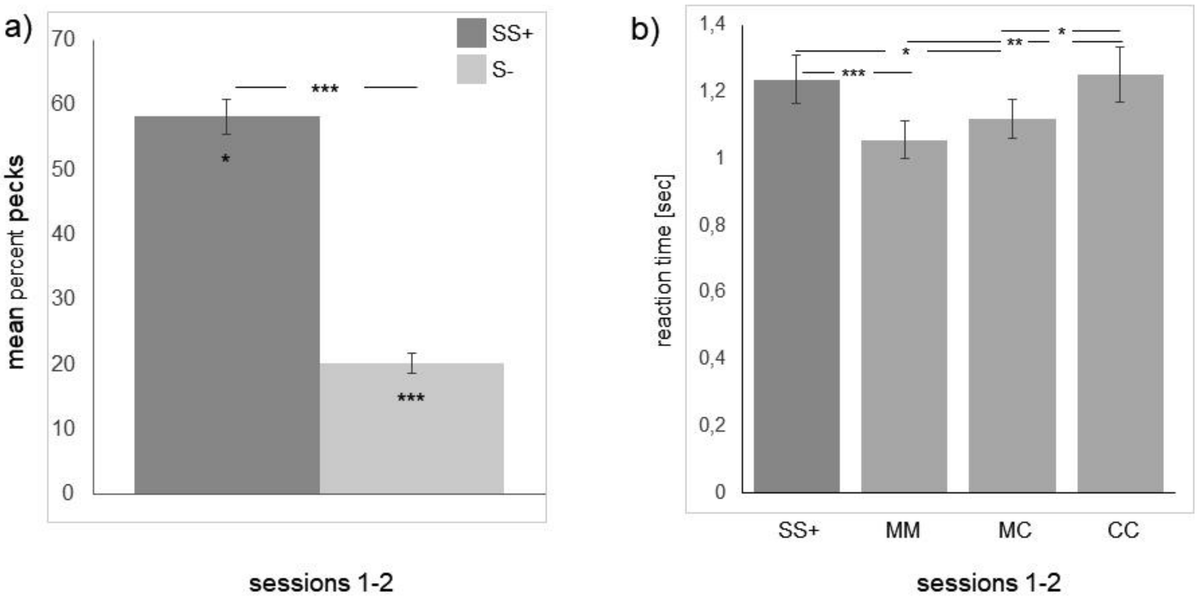

S1: Performances during the first two binocular conflict test sessions a) Percent pecks onto new positive (SS+) and negative (S-) stimuli. b) Response latencies for pecking onto SuperS+ (SS+) and conflict choices during binocular conflict tests (MM = Memory-based; CC = Conflict-based; MC = Memory- vs Category-based). Bars represent standard error (* $p < 0.05$; **, $p < 0.01$, *** = $p < 0.001$ according to t-tests for dependent samples or one sample t-tests).

Table S1: Correlation between different types of percent hemispheric-specific conflict decisions

	MM LH	MM RH	CC LH	CC RH	MC LH	CM LH	MC RH	CM RH
MM LH	1	-,423	,743**	0,033	,851**	,875**	-,125	-,096
MM RH	-,423	1	,020	,607*	-,143	-,031	,660*	,686*
CC LH	,743**	,020	1	-,083	,826**	,911**	-,150	-,023
CC RH	0,033	,607*	-,083	1	,028	,195	,790**	,809**
MC LH	,851**	-,143	,826**	,028	1	,856**	,028	-,182
CM LH	,875**	-,031	,911**	,195	,856**	1	-,004	,117
MC RH	-,125	,660*	-,150	,790**	,028	-,004	1	,719**
CM RH	-,096	,686*	-,023	,809**	-,182	,117	,719**	1

Pearson's *r* *correlation significant at level $p < 0.05$
Pearson's *r* **correlation significant at level $p < 0.01$
(LH = left hemispheric decision; RH = right-hemispheric decision, M= memory, C= category)

Table S2a: Correlation between left-hemispheric discrimination, or transfer performances and left-hemispheric conflict decisions

	M_LH	C_LH	MM_LH	CC_LH
M_LH	1	,166	,059	-,434
C_LH		1	,001	-,217
MM_LH			1	,743**
CC_LH				1

Pearson's *r* **correlation significant at level $p < 0.01$
LH = left-hemispheric performance or decision, M= memory, C= category

Table S2b: Correlation between right-hemispheric discrimination, or transfer performances and right-hemispheric conflict decisions

	M_RH	C_RH	MM_RH	CC_RH
M_RH	1	-,084	-,495	-,148
C_RH		1	-0,017	-,289
MM_RH			1	,607*
CC_RH				1

Pearson's *r* *correlation significant at level $p < 0.05$
RH = right-hemispheric performance or decision, M= memory, C= category

Table S2c: Correlation between asymmetry of monocular discrimination, or transfer performance and binocular choices

	AI M	AI C	AI MM	AI CC
	1	,116	,056	,509
AI C		1	,004	,135
AI MM			1	,468
AI CC				1

Pearson's *r*
AI asymmetry index, M= memory, C= category

Table S3a: Correlation between left-hemispheric choices and reaction times for different conflict types

	MM LH	CC LH	MC LH	CM LH	RT_MM LH	RT_CC LH	RT_MC LH	RT_CM LH
MM LH	1	,743**	,851**	,875**	-,029	,023	-,108	-,030
CC LH		1	,826**	,911**	,035	-,006	-,053	-,090
MC LH			1	,856**	-,042	,049	-,072	,019
CM LH				1	-,213	-,217	-,314	-,264

Pearson's *r* *correlation significant at level $p < 0.05$
Pearson's *r* **correlation significant at level $p < 0.01$
(LH = left hemispheric decision; RH = right-hemispheric decision, M= memory, C= category; RT = reaction time)

Table S3b: Correlation between right-hemispheric choices and reaction times for different conflict types

	MM RH	CC RH	MC RH	CM RH	RT_MM RH	RT_CC RH	RT_MC RH	RT_CM RH
MM RH	1	,607*	,660*	,686*	-,348	-,365	-,502	-,334
CC RH		1	,790**	,809**	-,726**	-,738**	-,777**	-,765**
MC RH			1	,719**	-,326	-,331	-,410	-,399
CM RH				1	-,559	-,599*	-,678*	-,635*

Pearson's *r* *correlation significant at level $p < 0.05$
Pearson's *r* **correlation significant at level $p < 0.01$
(RH = right-hemispheric decision, M= memory, C= category; RT = reaction time)

Table S4: Stimuli Sets / Test

Experimental phase	Stimulus sets	Stimulus Composition/ Session
Training	4x50 S+/ 4x50 S- (dog category)<br>4x50 S+/ 4x50 S- (cat category)	100 % positive (S+)/ negative (S-) Training Stimuli
Transfer	4x50 S+/ 4x50 S- (dog category)<br>2x 30 TS+/ TS- (dog category)<br>4x50 S+/ 4x50 S- (cat category)<br>2x 30 TS+/ TS- (cat category)	80% positive / negative Training Stimuli<br>20% positive / negative Transfer Stimuli
Conflict	45 SS+/45 SS-<br>4x 2x 15 TD/TC<br>2x 15 UTD/UTC<br>4x 2x 15 TD/UTC<br>4x 2x 15 UTD/TC	60% positive / negative Super Stimuli<br>10% Trained Dog/ Trained Cat stimulus pairs<br>10% UnTrained Dog / UnTrained Cat stimulus pairs<br>10% Trained Dog / UnTrained Cat stimulus pairs<br>10% UnTrained Dog / Trained Cat stimulus pairs

(For Training, four parallel stimulus sets were compiled presenting comparable settings; For Transfer and Conflict tests, stimuli were grouped in two parallel sets, which were presented alternately)

Table S5: Trials composition of the different experimental phases

Test phase	Composition of one session	Test Repetition
Monocular Training	300 Training Trials	Until learning criterion with each eye
Monocular Transfer	120 Transfer + 180 Training Trials	8-12
Binocular Conflict choice	120 Conflict + 180 SuperS+/S- Discrimination Trials	12-16
